# 

**Published:** 1847-03

**Authors:** 


### WESTERN JOURNAL

OF

### MEDICINE AND SURGERY.

### EDITORS.

---

DANIEL DRAKE, M.D.,

PROFESSOR OF PATHOLOGY AND THE PRACTICE OF MEDICINE IN THE MEDICAL INSTITUTE OF LOUISVILLE:

LUNSFORD P. YANDELL, M.D.,

PROFESSOR OF CHEMISTRY AND PHARMACY IN THE SAME:

THOMAS W. COLESCOTT, M.D.

### RECEIPTS OF MEDICAL JOURNAL FOR JANUARY AND FFBRUARY.

| | |
|---|---|
| Dr. W. Smith, Newtown, Ohio, | $2 00 |
| Dr. Shuford, Waterfod, Miss., | 5 00 |
| E. H. Bruce, Peru, Ind., | 35 00 |
| R. T. Thornton, Independence, Mo., | 10 00 |
| G. L. McAfee, Elizabethtown, Ky., | 10 00 |
| J. S. Helms, Tuscumbia, Ala. | 5 00 |
| J. C. Kirkpatrick, Stewartsboro', Tenn., | 15 00 |
| C. F. Brown, Van Buren, Ark., | 5 00 |
| H. B. Stubblefield, Alexandria, Ala., | 5 00 |
| B. Kerrick, Jefferson co., Ky., | 10 00 |
| —— Robinson, " " | 5 00 |
| D. H. Davis, Jackson, Mo., | 5 00 |
| J. Brooks, Eggs Point, Miss., | 5 00 |
| J. Edwards, Leavenworth, Ind., | 5 00 |
| R. Steele, Paris, Ill., | 5 00 |
| J. G. Brian, Wilsonville, Ky., | 5 00 |
| A. M. Scruggs, Eastport, Miss., | 5 00 |
| J. Travis, Martins Creek, Tenn., | 5 00 |
| W. F. Miller, Owensboro', Ky., | 5 00 |
| H. F. Abell, West Point, Ky., | 20 00 |
| L. B. Hobbs, Reeves, Mo., | 5 00 |
| G. W. Wheeler, Perryville, Mo., | 5 00 |
| A. S. Wharton, Prairie Point, Miss., | 5 00 |
| J. Slavins, Portland Mills, Ind., | 5 00 |
| E. O. Brown, Brandenburg, Ky., | 10 00 |
| E. Geoghegan, Elizabethtown, Ky., | 10 00 |
| W. Williams, Nashville, Tenn., | 5 00 |
| J. P. Henderson, Newville, O., | 4 47 |
| J. M. Quinn, Brandon, Miss., | 25 00 |
| G. O. Pond, Columbus, Ill., | 5 00 |
| G. R. McCoy, Golconda, Ill., | 20 00 |
| P. Winfree, New River, La., | 5 00 |
| L. B. Robinson, Murfreesboro', Tenn., | 5 00 |
| —— Shanks, Memphis, Tenn., | 5 00 |
| J. Roberts, Montezuma, Ind., | 5 00 |
| J. Thompson, New Geneva, Pa., | 20 00 |
| A. Moore, Memphis, Tenn., | 5 00 |
| O. Mitler, Monticello, Ky., | 5 00 |
| W. A. Hickman, Bardstown, Ky., | 10 00 |
| U. P. Golliday, Robin's Nest, Ill., | 10 00 |
| L. L. Tolson, California, Miss., | 5 00 |
| E. Andrews, Liberty Hill, Miss., | 3 00 |
| G. A. Martin, Salem, Miss., | 5 00 |
| W. H. Lee, Mexico, Mo., | 5 00 |
| H. P. Griswold, Plymouth, Ill., | 10 00 |
| W. Smith, Newtown, O., | 3 00 |
| Wilson & Smith, Jefferson, Tenn., | 5 00 |
| S. A. McKay, Shepherdsville, Ky., | 5 00 |
| M. H. Harding, Lawrenceburg, Ind., | 5 00 |
| T. L. Armstrong, Athens, Miss., | 13 00 |
| D. Smith, Brickville, Ala., | 5 85 |
| W. G. Montgomery, Independence, Ind., | 10 00 |
| P. S. Secor, Bernadotte, Ill., | 5 00 |
| Van Eaton, Mt. Hope, Ala., | 20 00 |
| R. Robson, New Harmony, Ind., | 10 00 |
| A. C. Lewis, Scott, O. | 10 00 |
| H. Brown, Iowa City, Iowa, | 10 00 |
| J. C. Bernard, Liberty, Mo., | 5 00 |
| J. G. Lowry, Helena, Ark., | 15 00 |
| —— S. Stanfield, Warren, Ark., | 20 00 |

### TO DELINQUENT SUBSCRIBERS.

[illegible]t the commencement of the year, we struck from the subscription list of this [illegible]ournal the names of 117 subscribers, who owed us an aggregate of over $3,000, or about $30 apiece. The accounts of these gentlemen had repeatedly been presented by letter and by agents, and in discontinuing them we addressed them a letter potent enough to extort from the greater part of them our dues. Those who make no reply may fairly be regarded as void of shame as well as of honesty; but this consideration will not prevent our dealing with them, in due time, according to the letter of the circular.

We do hope that those who remain on our list, will treat us with more fairness. We trust that they will not wait for agents or letters, but simply remit us what they owe us. The relation is one of trust. We do not know our subscribers, and in receiving our publication, they incur obligations in the nature of a debt of honor, against which no plea can be received. Gentlemen, please all remit at once. [March 1—2t] PRENTICE & WEISSINGER.

---

☞ Our ample experience of the difficulty and uncertainty of collecting from distant subscribers, compels us to decline all orders for this Journal not accompanied by the cash or a satisfactory reference. We were reluctant to adopt this course, but success in our undertaking requires it, and it can result in no material inconvenience to subscribers. All remittances by mail, post paid, are at our risk.

Feb 1847 PRENTICE & WEISSINGER.

The WESTERN JOURNAL OF MEDICINE AND SURGERY is published monthly by the undersigned, at the corner of Main and Fifth streets, Louisville, at $5 per annum, payable in advance. Each number contains 96 pages, making two volumes in the year of more than 550 pages each.

Contributions to its pages by the Physicians of the Valley of the Mississippi, addressed to the Editors, are respectfully solicited.

Letters on business, to be addressed, postage paid, to the publishers.

*January*, 1844. PRENTICE & WEISSINGER.

